# Jute fiber-derived functional cellulose nanocrystals for efficient methylene blue dye adsorption

**DOI:** 10.1039/d5ra09124a

**Published:** 2026-07-03

**Authors:** Md. Fahimuzzaman, Md Shahabul Hossen, Tarikul Islam, Shanzida Binte Hassan, Khairul Islam, M. Mahbubul Bashar

**Affiliations:** a Department of Textile Engineering, Mawlana Bhashani Science and Technology University Santosh Tangail 1902 Bangladesh bashar.te@mbstu.ac.bd; b Department of Textile Merchandising and Interiors, University of Georgia Athens Georgia 30602 USA tarikul@uga.edu; c Department of Textile Engineering, Jashore University of Science and Technology Jashore 7408 Bangladesh

## Abstract

Adsorption has become the most familiar approach for removing dye from textile effluent. Achieving high dye removal efficiency *via* inexpensive, sustainable adsorbents is a common dilemma. In recent years, highly functional cellulose nanoparticles have been a promising candidate for dye adsorption. This study employed functional cellulose nanocrystals (FCNCs), isolated from jute fiber using the oxidization method, as an adsorbent to remove methylene blue (MB) from an aqueous solution. The different characteristics of FCNCs were analyzed with thermogravimetric analysis (TGA), X-ray diffraction (XRD), and Fourier-transform infrared spectroscopy (FTIR). The FTIR analysis revealed that the oxidation of jute fiber by ammonium persulfate (APS) produced a carbonyl peak at 1730 cm^−1^. The TGA study showed a single peak between 305 °C and 318 °C, indicating good thermal properties. The XRD graph demonstrated FCNCs were highly crystalline, with a crystallinity index of 70%. The impacts of pH, temperature, and initial dye concentration were explored to maximize the amount of dye adsorption. The adsorption equilibrium data was analyzed with the Langmuir and Freundlich isotherm models; however, the Langmuir model provided a more accurate description of the adsorption procedure. The highest possible adsorption capability at room temperature and unadjusted pH (pH = 6.4) was 1566.95 mg dye per g FCNCs. This result suggests that FCNCs are apparently an effective adsorbent for removing cationic dyes.

## Abstract 2

Ammonium persulfate oxidized jute-based functional cellulose nanocrystals, which exhibited high crystallinity and greater thermal stability, enabling high methylene blue dye adsorption, indicating suitability for wastewater treatment.

## Graphical abstract

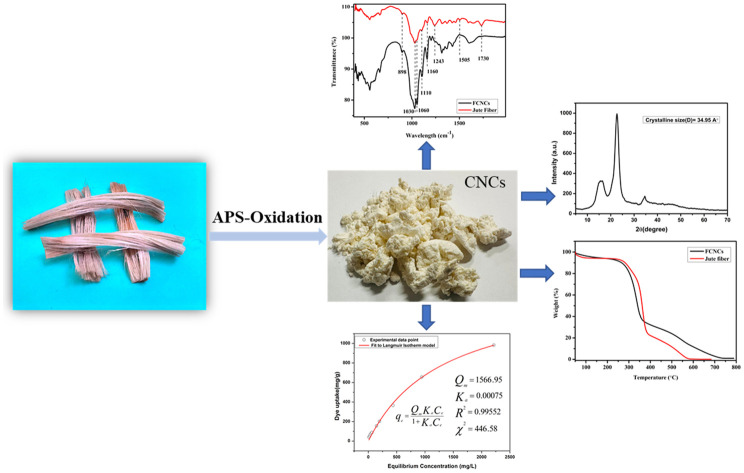

## 1. Introduction

Dyes and other chemicals contaminating the environment are mostly released into the wastewater from textile manufacturing processes and pharmaceuticals. Dyes are aromatic organic compounds employed in the textile manufacturing. They are generally categorized into three groups: anionic, non-ionic, and cationic. Anionic and cationic dyes are most employed in the textile industry due to their smooth application, good water solubility, and relatively inexpensiveness.^1,2^ Eliminating cationic dyes from water has received greater attention than removing anionic dyes because cationic dyes are more easily absorbed by cells and can interact with charged semipermeable membrane surfaces, leading to severe health issues. Methylene blue (MB) is a popular cationic dye that is commonly used for dyeing wool, silk, and cotton fibres.^3,4^ Pollution from MB causes serious health problems like methemoglobinemia, respiratory problems, nausea, vomiting, and eye burns.^5,6^ Since they show potential toxicity to human life and ecosystems, it is, therefore, essential to remove them during wastewater treatment. However, a variety of treatment methods have been developed for the removal of dyes. These include chemical, biological, and physical methods.^7^ Adsorption is one of them, and it has been considered better than other traditional methods for environmental and economic reasons. These reasons include its effectiveness, minimal initial investment, ease of installation and handling, and sensitivity to harmful compounds.^8^ Moreover, adsorption gives the best outcomes and, with the proper design, may generate treated water of the highest caliber.^9^ There are various adsorbents have been extensively used for MB adsorption, such as cellulose nanocrystals (CNCs), activated carbon, silica, clays, nanoparticles, graphene oxide (GO), carbon nanotubes, alginate-GO composite gels, surface-modified cellulose, and a variety of agricultural wastes such as sawdust and fruit peels.^5,10–13^ Considering its high functionality and large surface area, activated carbon shows high adsorption capacity for removing MB. However, they are not as economically viable and available as cellulose. On the contrary, cellulose nanocrystals are the most effective materials widely available on Earth. Various kinds of biomass can be active sources of cellulose. Among them, jute is a potential source of cellulose; it contains a higher amount of cellulose; almost 65% of cellulose is obtained from jute fiber, and its annual worldwide production is roughly 3 million metric tonnes, approximately equal to the production of cotton fiber. Jute can grow on any land with little or no fertilizer or pesticide. By contrast, flax, hemp, sisal, and ramie contain cellulose nearly equal to or more than jute fiber, but their annual production rate is very low. FCNCs can be obtained by different techniques, like mechanical, chemical, and enzymatic processes.^14,15^ CNCs are synthesized by a variety of chemical processes; sulfuric acid hydrolysis is one of the most common techniques. The significant drawbacks of this process are that it is challenging to handle highly concentrated sulfuric acid and produce CNCs containing a sulfate half-ester group. Because they contain a sulfate half-ester group, CNCs made with sulphuric acid hydrolysis are very good at dispersing in water but not so good at withstanding high temperatures. On the other hand, ammonium persulfate (APS), an effective oxidizer, has recently been used in the extraction of (FCNCs). It has several advantages, such as low long-term toxicity, excellent solubility in water, cost-effectiveness, and ease of handling. Oxidation produces free radicals that decolorize biomass and break down CNCs at the same time. These free radicals are called persulfate and peroxide. Due to oxidation, it generates carboxylate ions at the C-6 position of cellulose, which is more functional and stable than the sulphate half-ester group. Furthermore, handling them is far safer than handling extremely concentrated sulfuric acid. 2,2,6,6-Tetramethylpiperidine-1-oxyl radical (TEMPO)-mediated oxidation is a popular technique of producing a carboxylate group containing cellulose.^16^ Yet, these reports are based on a variety of chemical processes that involve pre-treatment, bleaching, and isolation that involve numerous harmful reagents and chemicals. In this sense, the bath procedure makes the APS oxidization process relatively easy to produce FCNCs.^17^

This work uses the oxidization process to synthesize FCNCs from jute fiber, which is then used for the removal of MB from aqueous solutions. The maximal dye uptake capacity of FCNCs is determined by investigating the impact of the initial dye concentration. The investigation also focuses on the effects of pH, temperature, and removal by FCNCs.

## 2. Experimental section

### 2.1 Reagents and materials

The jute fiber was collected from a Bangladeshi market and used as the initial material for isolating FCNCs. Purified ammonium persulfate (APS) was procured from SIGMA-ALDRICH. Co. The analytical-grade chemicals ethanol, hydrochloric acid (HCl) solutions, sodium hydroxide (NaOH), and organic solvents were all employed without purification.

### 2.2 Synthesis of FCNCs

**Fig. 1:**
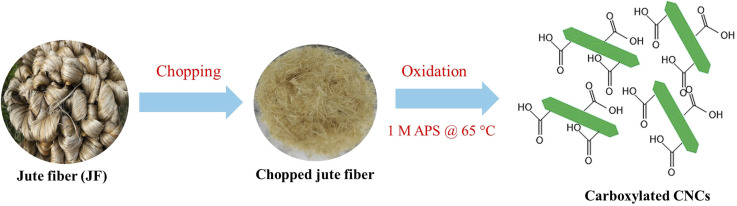
Schematic diagram of synthesis of FCNCs.

Jute fibers were chopped to a length of 2″–3″ using scissors, followed by washing with acetone and ethanol for 10 min. The chopped jute fiber (2 g) was mashed with a commercial blender. The jute fiber was then added to a conical flask containing 100 mL of deionized water and 22.82 mg (1 M) of APS. The system was boiled at 125 °C and 1.25 kPa for 1 hour. After boiling, the mixture was cooled to room temperature and another 100 mL of 1 M APS solution was added to the previous solution. It was then stirred for 16 hours with a thermo-controller between 60 and 65 °C. Afterwards, a Buchner funnel was used to vacuum-filter the suspension, which was then drained of deionized water after a specified reaction time to reach pH neutral. A membrane filter was used to dialyze the FCNCs suspension for at least 24 hours. The raw FCNCs were obtained and vacuum-dried by vacuum filtering for the entire night. Finally, powdery FCNCs were stored in a vacuum chamber for further investigation. Fig. 1 exhibits the overview of synthesis of FCNCs.

### 2.3 Characterization

Fourier transform infrared spectroscopy was performed using Nicolet IS20 FTIR analyzer with KBr/Ge beam splitter to analyze the surface chemical properties of the samples over the wavelength range of 500–4000 cm^−1^.^18^ Thermogravimetric analysis (TGA) was employed to investigate thermal properties of the samples. This test was carried out using a TGA4000 apparatus (Brand: Parkin Elmer).^19^ It was performed at 50 °C to 800 °C with a heat rate of 10 °C min^−1^ while N_2_ gas flowed continuously. A crystallographic analysis of the samples was performed using an X-ray diffractometer (Model: SmartLab, Manufactured in Japan).^20^ This instrument was conducted at 40 kV voltage and 40 mA power, and the XRD pattern was generated using a Cu-kα X-ray beam. The crystallinity index (CI) was determined by eqn (1).^14^1
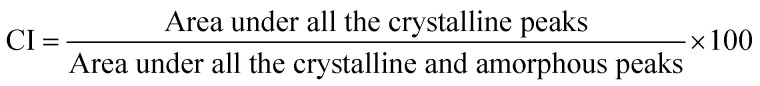


**Fig. 2:**
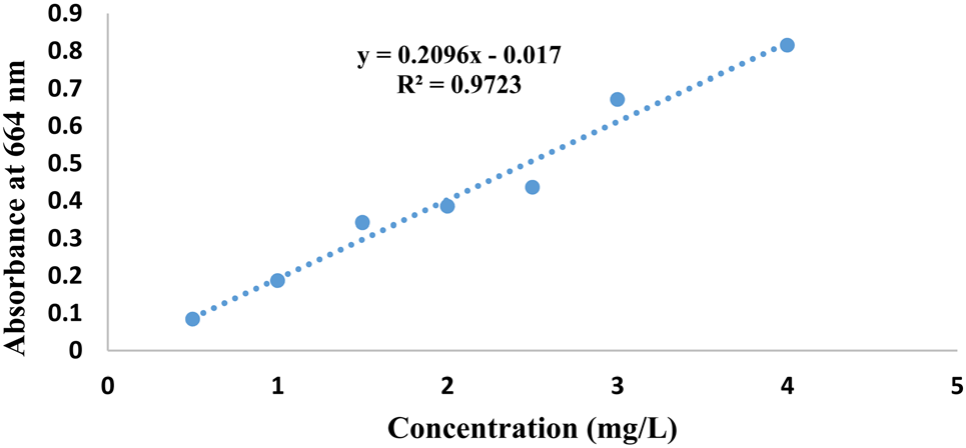
MB calibration curve.

Adsorption experiments were performed by preparing stock solutions of MB at concentrations ranging from 50 to 3200 ppm. Then, 10 mL of each MB solution and 10 mg of adsorbent (FCNCs) were mixed into different 25 mL vials. After that, the vials were shaken at ambient temperature and 120 rpm for 60 min. After that, the vials were removed from the shaker bath and allowed to rest for 10 minutes. Then, diluting these solutions at 20–1280 times for maintaining a common concentration of 2.5 ppm, because at higher concentrations like 50 ppm, the absorbance will be greater than 1. The concentration of MB was determined by UV-vis spectroscopy. The calibration curve shown in Fig. 2 is used to convert absorbance data to dye concentration.

The quantity of dye adsorbed on FCNCs surface was determined using eqn (2):2
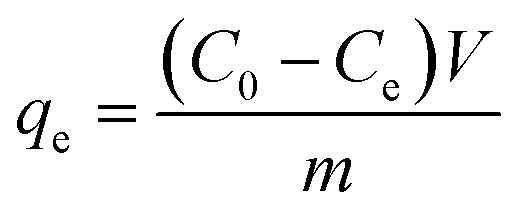
In this equation, *q*_e_ represents the amount of dye adsorbed per gram of adsorbent (mg g^−1^), *C*_e_ represents the dye concentration at equilibrium (mg L^−1^), *C*_0_ represents the initial dye concentration (mg L^−1^), *V* indicates the dye solution volume (L), and *m* is the mass of adsorbent (g).

The dye removal percentage was computed using eqn (3):3
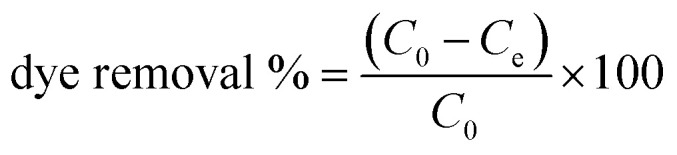


An adsorbent dosage of 10 mg was used for every 10 mL of dye solution to examine the effect of initial dye concentration, temperature, and p^H^. The effect of initial dye concentration was examined by varying the initial concentration of MB from 50 to 3200 mg L^−1^, while maintaining a constant pH (6.4) and temperature at room conditions. This study investigated the effects of temperature variation between 30 °C and 90 °C, while maintaining a dye concentration of 50 mg L^−1^. The impact of pH on dye adsorption by cellulose nanocrystals was also investigated by conducting dye adsorption experiments at five distinct pH values—2, 4, 6, 8, and 10—at room temperature and a dye concentration of 50 mg L^−1^.

## 3. Results and discussion

### 3.1 Surface chemistry of FCNCs

**Fig. 3:**
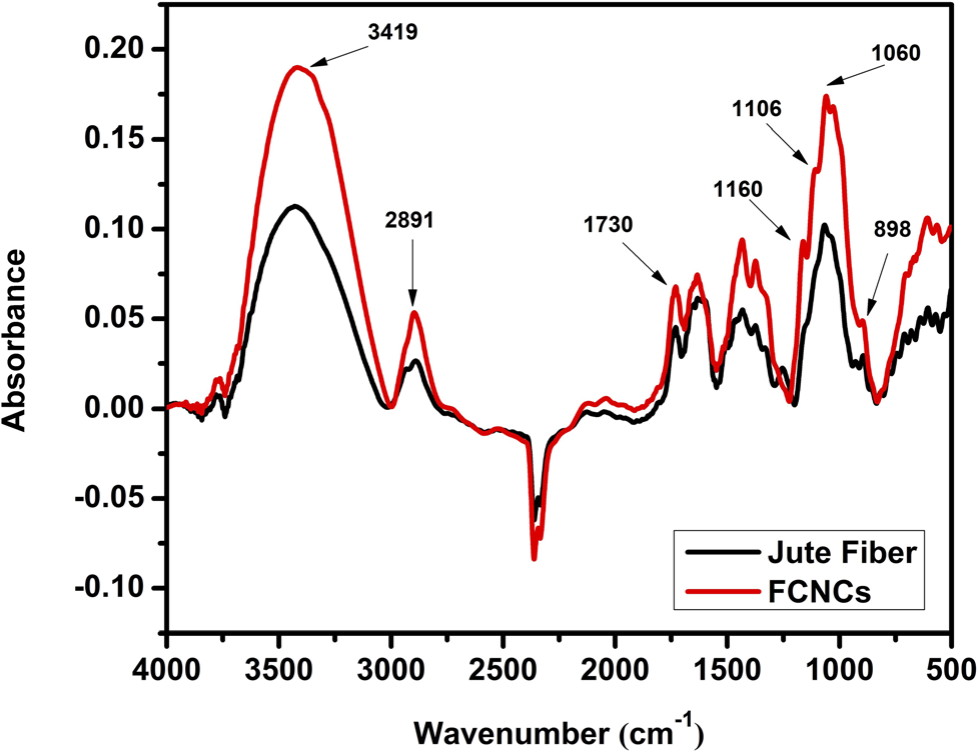
FT-IR spectrum of FCNCs derived from jute fiber.

FTIR was employed to analyze the surface functional groups of FCNCs produced by APS oxidation (Fig. 3). The FCNC spectrum exhibited a prominent peak between 3600 and 3000 cm^−1^, corresponding to O–H stretching vibrations. Additionally, peaks related to the stretching and bending vibrations of aliphatic C–H and CH_2_ groups are observed at 3000–2800 cm^−1^ and 1500–1250 cm^−1^, respectively. Distinctive peaks at 898, 1060, 1106, and 1160 cm^−1^ confirmed the presence of skeletal C–O–C bonds in glucose and pyranose rings, indicating that nearly pure cellulose was extracted and that the oxidation process did not significantly alter the structural framework of cellulose.

Compared to jute fiber, these peaks were less pronounced than those of FCNCs. This was relevant because non-cellulosic polysaccharides, such as lignin, hemicellulose, and wax cemented jute fiber. The key point was that APS oxidized the jute fiber, leading to a prominent carbonyl (C=O) stretching peak at around 1730 cm^−1^ due to carboxylation.^15,21,22^ In contrast, lignin and the hemi-cellulosic acetyl or uronic ester groups were shown to be the source of a peak at 1728 cm^−1^ in raw jute fiber.^23,24^ During the early oxidation period with APS, the carbonyl peak at 1728 cm^−1^ decreased, and a new peak at 1730 cm^−1^ appeared as oxidation continued, with the oxidation duration increasing to 8 to 16 hours. This evidence for the reformation of the carboxyl group was discussed in detail elsewhere by Bashar *et al.*^15^ The presence of the carboxyl group on the FCNC surface was further corroborated through conductometric titration, which quantified the –COOH groups. The results indicated a generation of 1470 mmol kg^−1^ of –COOH groups, accompanied by a corresponding zeta potential of −40 mV.^15^

### 3.2 Thermal stability of FCNCs

**Fig. 4:**
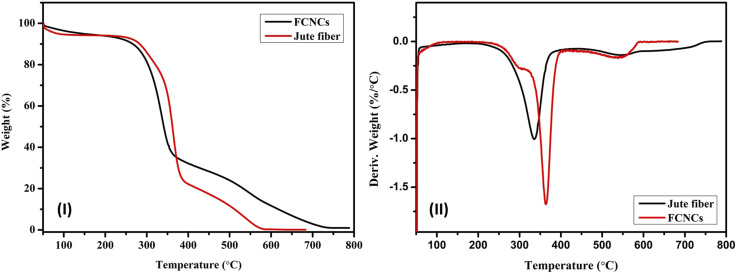
Thermal characteristics: (I) TGA and (II) DTG profiles of FCNCs and jute fiber.

Fig. 4 displays the TGA and DTG patterns of FCNCs and jute fiber. The degradation of cellulose is initiated with depolymerization, dehydration, and the simultaneous breakdown of the glycosyl unit.^15,25–27^ While jute fiber began to decay at 270 °C, the FCNCs showed signs of degradation at 215 °C, indicating lower thermal stability of FCNC than the parent jute fiber. Likewise, it was found that the maximum temperature at which FCNCs became decomposed was 337 °C, which is lower than the 342 °C temperature of jute fiber. Consequently, the low thermal stability of the APS-oxidized FCNCs gives better findings from other investigations of carboxylic group-decorated cellulose nanoparticles.^21,22^

Compared to raw jute cellulose, this behaviour may be explained by the smaller size of the particle and larger specific surface area, leading to lower heat resistance and active surface groups.^22^ FCNCs, however, reached temperatures approximately 100 °C higher than jute fibres at 5% weight loss, as raw jute fibre contains low-molecular-weight non-cellulosic components that decompose more quickly. Fig. 4(II) depicts the DTG profile of jute fibre, showing three local maxima at 285, 342, and 451 °C. In that order, these maxima correspond to weight losses of 13.5, 51.8, and 87%. These maxima represent the pyrolysis of cellulose, crystalline cellulose, and the breakdown of lignin and hemicellulose. By contrast, the FCNCs showed just single peaks between 305 and 318 °C, suggesting that crystalline cellulose was produced in a highly pure form.

### 3.3 Crystal structure of FCNCs

**Fig. 5:**
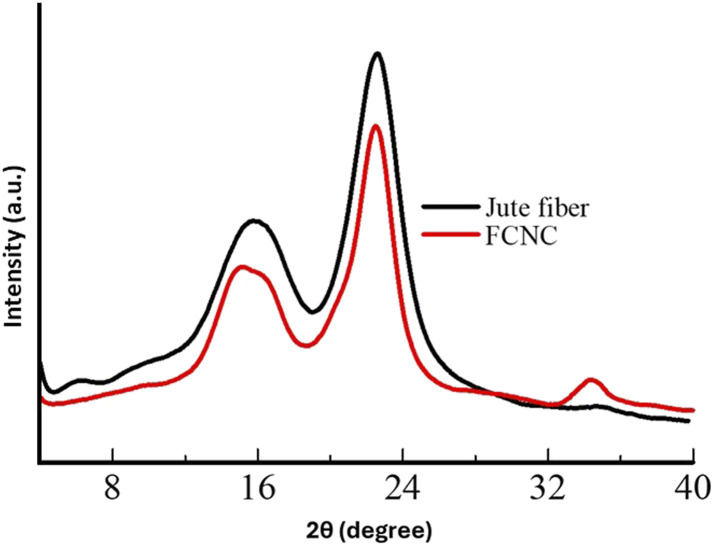
XRD analysis of jute fiber and FCNCs.

The study employed X-ray diffraction (XRD) to examine the crystal structure of raw jute fiber and FCNCs derived from APS-oxidized jute fiber (Fig. 5). The FCNCs display peaks at 2*θ* values of 14.6°, 16.4°, 22.5°, and 34.4°, which indicate a cellulose I structure. In contrast, these peaks are less prominent in raw jute due to the presence of noncellulosic polysaccharides, aligning with FT-IR findings. A peak at 22.5° confirms native cellulose crystallinity. Raw jute exhibits approximately 60% crystallinity, while FCNCs have a similar level to raw jute. However, the crystallinity of FCNCs were nearly 70%, attributable to the removal of noncellulosic polysaccharides and the breakdown of amorphous regions during APS treatment. Slight oxidation further reduces crystallinity, as prolonged reaction times can damage crystalline regions.

### 3.4 Dye adsorption performance

#### 3.4.1 Effect of initial dye concentration

**Fig. 6:**
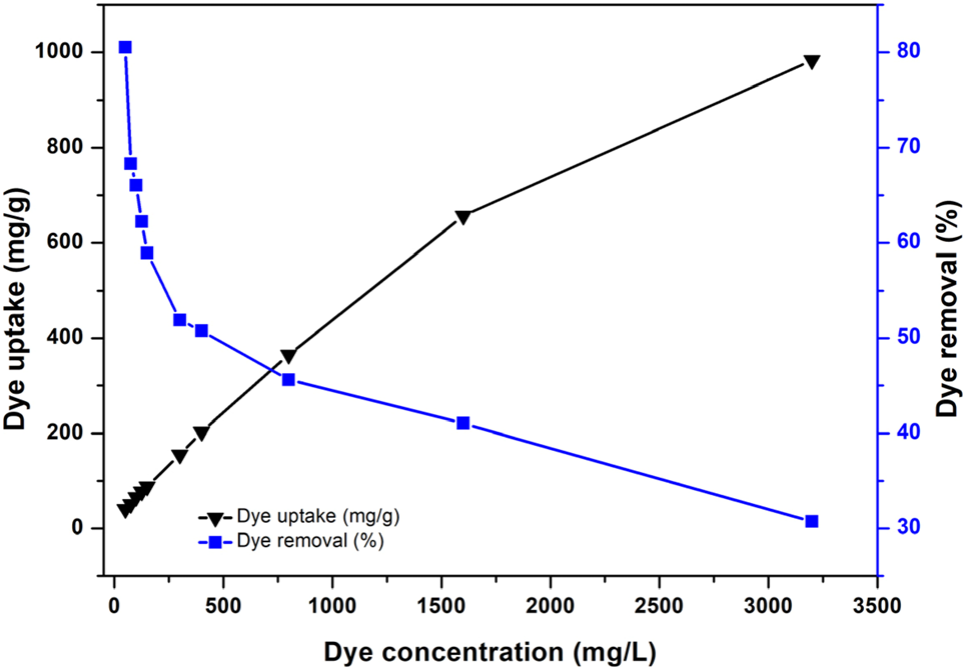
Impact of initial dye concentration on dye uptake and dye removal by FCNCs.

Fig. 6 displays the impact of dye concentration on dye uptake (*q*_e_) and dye removal (%) by FCNCs. It was observed that the dye adsorption capacity improved with an increase in the initial dye concentration (*C*_0_) for a fixed quantity of absorbents (10 mg). This is due to the increased driving force between the adsorbents and dye as the initial concentration increases. In contrast, as dye concentration (*C*_0_) increases, the availability of adsorption sites decreases, leading to decrease in the dye removal percentage. In addition, this phenomenon is logical since the ratio of increased dye concentration to increased dye adsorption decreases as the dye concentration increases.

Fig. 6 also presents two totally different curves, which means that the dye removal percentage and dye uptake percentage are different. This is because when the dye concentration in the solution is lower, the adsorption sites in the fibre are not yet saturated. However, as the concentration increases gradually, the fibre utilises its maximum adsorption sites to adsorb the dye. As a result, the dye removal percentage decreased while the uptake percentage increased.

#### 3.4.2 Effect of temperature

**Fig. 7:**
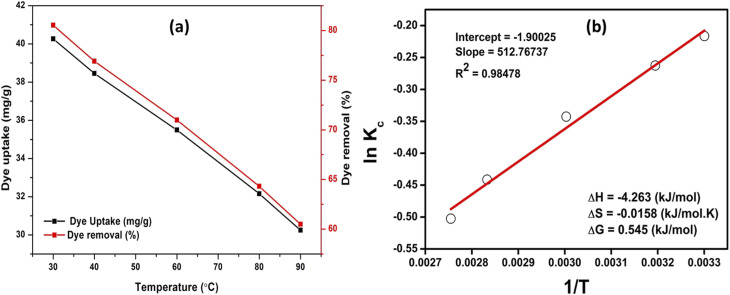
(a) Impact of temperature on dye uptake and percentage of dye removal by FCNCs, (b) the natural logarithm of partition coefficient ln *K*_c_ against 1/*T*.

The percentage of dye removal and adsorption against temperature is illustrated in Fig. 7. This figure shows that dye removal decreased by approximately 30% at higher temperatures (90 °C), while lower temperatures supported higher adsorption, confirming the exothermic nature of the adsorption process. To elucidate the exothermic nature, the following formulae were employed to calculate the thermodynamic parameters of adsorption: enthalpy (Δ*H*), entropy (Δ*S*), and free energy (Δ*G*).^28^

4 Δ*G* = −*RT* ln *K*_c_

5 Δ*G* = Δ*H* − *T*Δ*S*

Based on eqn (4) and (5),−*RT* ln *K*_c_ = Δ*H* − *T*Δ*S*6
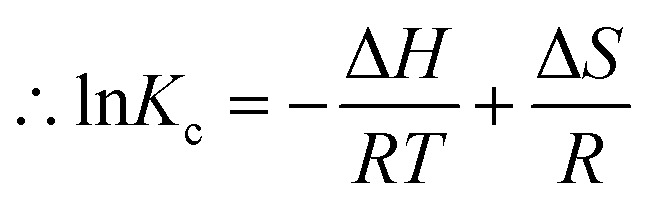
In this case, *T* represents the temperature on the Kelvin scale (*K*), and *R* is the gas constant (J kmol^−1^). *K*_c_ is the adsorption partition coefficient, which represents the ratio between dye concentration on the adsorbent and dye concentration in solution under equilibrium conditions. In Fig. 7, the natural logarithm of the partition coefficient (ln *K*_c_) was plotted against 1/*T*, the inverse of temperature. Based on this figure, eqn (6) assumes that the data roughly reflect a linear relationship with a correlation coefficient of 0.984.

Based on the slope and intercept of the fitted line within this figure, the change in the thermodynamic attributes of adsorption, enthalpy, and entropy was calculated as Δ*S* = −0.0158 kJ mol^−1^ K^−1^ and Δ*H* = −4.263 kJ mol^−1^, respectively. Considering the negative sign of the change in enthalpy, the adsorption process is exothermic. This supports our findings in Table 1 and explains why dye removal decreased as the temperature increased. Lastly, the equation was used to calculate the change in free energy (Δ*G*) at a given temperature. Δ*G* fluctuated between 0.545 kJ mol^−1^ at 30 °C and 1.516 kJ mol^−1^ at 90 °C within the examined temperature range.

**Table 1:** Thermodynamic parameters related to MB adsorption on FCNCs

Temperature (°C)	*K*_c_	Δ*G* (kJ mol^−1^)	Δ*H* (kJ mol^−1^)	Δ*S* (kJ mol^−1^ K^−1^)
30	0.805	0.545	−4.263	−15.798
60	0.709	0.948	−4.263	−15.798
90	0.605	1.516	−4.263	−15.798

#### 3.4.3 Effect of P^H^

**Fig. 8:**
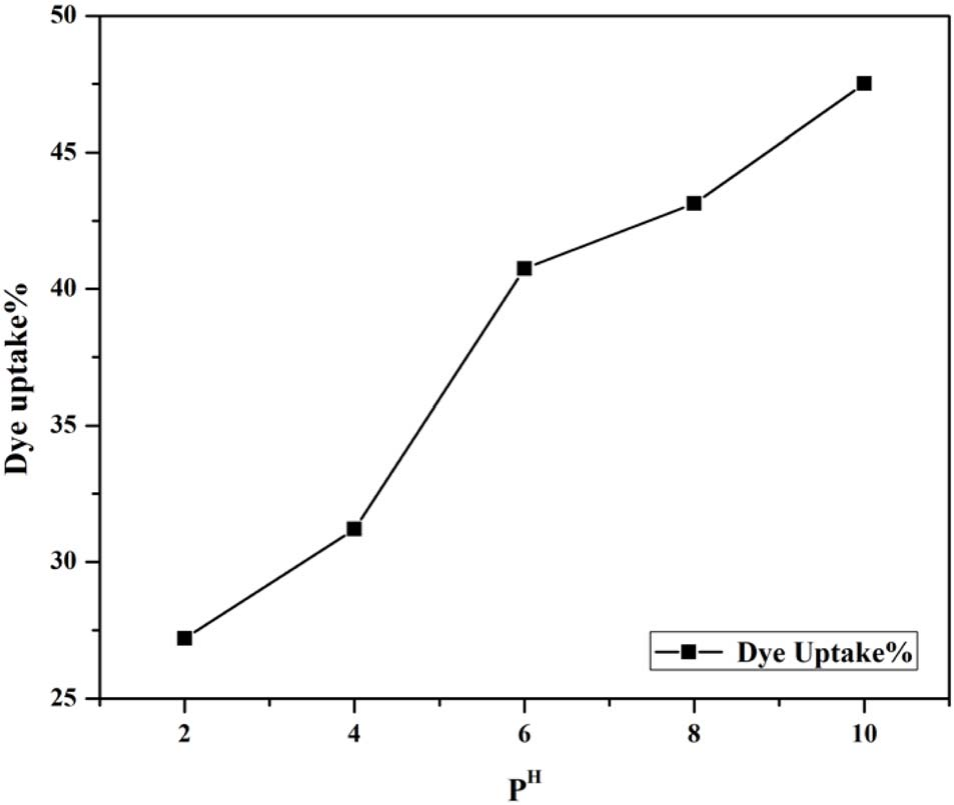
Effect of p^H^ on dye uptake.

The stock solution of 50 mg MB had a pH of 6.4. The pH of the solution was adjusted with HCl and NaOH. Fig. 8 displays the impact of p^H^ on the adsorption of MB by FCNCs. It also indicates that the dye uptake percentage increases with a high pH value.

The adsorption capacity of the adsorbent greatly depends on the pH value of the dye solution; thus, it can influence the surface charge of the adsorbent as well as the dye in an aqueous medium *via* the protonation and deprotonation of functional groups.^4,29^ At low pH, the protonation of functional groups such as carboxylic acid occurs, leading to a decline in surface charge and competition between the proton and the binding sites.^6,30^ Many studies already attribute the removal capacity of positively charged dyes and heavy metal cations to cellulose, which becomes low at low pH.^31–34^ In addition, the charged hairs on FCNCs may collapse on themselves at low pH values or strong ionic strengths due to the lack of electrostatic stability, which lowers the accessibility of binding sites.^35^

### 3.5 Adsorption isotherms

**Fig. 9:**
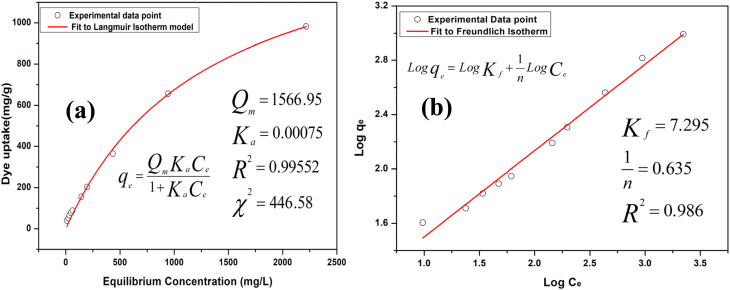
(a) Non-linear analysis of the Langmuir and (b) linear analysis of the Freundlich isotherm model.

Adsorption isotherms describe an interaction between the concentration of liquid-phase dye at constant temperature and the dye adsorbed per unit of sorbent mass. Thus, Fig. 9 depicts the MB adsorption onto FCNCs as a function of dye solution equilibrium concentration (*C*_e_, mg L^−1^). This study used two standard isotherm equations, the Freundlich and Langmuir isotherms, to determine the isotherm parameters.

The Langmuir isotherm describes a surface with uniform affinity through the sorption site. This isotherm also describes the equilibrium between the adsorbent and adsorbate systems. The Freundlich isotherm is used to describe a surface for multilayer adsorption on heterogeneous sites. To evaluate the adsorption isotherm of the MB on FCNCs, based on the above equilibrium data, the following equations (eqn (7) and (8)) can be used to express the Langmuir equation and the Freundlich isotherm models:7
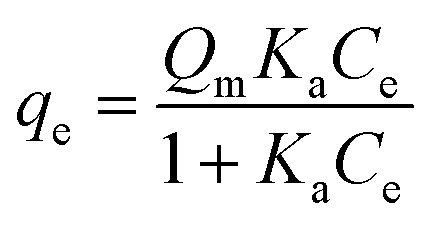
8
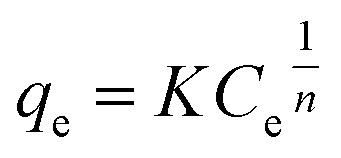
In this equation, *Q*_m_ is the greatest amount of dye that can be adsorbed per gram of adsorbent (mg g^−1^) and *q*_e_ is the quantity of dye adsorbed per gram of adsorbent (mg g^−1^) at equilibrium. The equilibrium concentration of free dye molecules in the solution is denoted by *C*_e_ (mg L^−1^). At the same time, *K*_a_ (L mg^−1^) and *K* (L mg^−1^) are the parameters associated with the capacity of adsorption, indicating the affinity between the dye and adsorbent, the heterogeneity factor (1/*n*), which varies from 0 to 1.

An assessment of an isotherm equation is based on the correlation coefficient, *R*^2^. Fig. 9 illustrates Langmuir and Freundlich's isotherm models for MB adsorption on FCNCs. Summarize the parameters of different forms of isotherm equations for the MB adsorption on FCNCs in Table 2.

**Table 2:** The isotherm parameter for FCNCs

Dye	Langmuir			Freundlich		
	*Q*_m_ (mg g^−1^)	*K*_a_ (L mg^−1^)	*R* ^2^	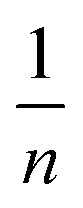	*K*_f_	*R* ^2^
MB	1566.95	0.00075	0.99552	0.635	7.295	0.986

Table 2 presents correlation coefficients, indicating that the Langmuir model (*R*^2^ = 0.99552) provides a significantly better fit to the MB adsorption of FCNCs than the Freundlich model (*R*^2^ = 0.986). This suggests that MB adsorption occurs on FCNCs that are energetically equivalent and homogeneous. Moreover, the results demonstrate that dyes do not interact with or transmigrate to nearby surface planes. The feasibility and suitability of the adsorption process are determined using the dimensional separation factor, *R*_L_.^36^
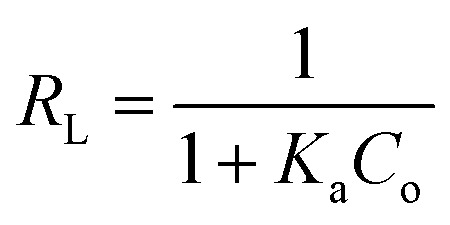
where *C*_o_ is the initial concentration in solution (mg L^−1^) and *K*_a_ (L mg^−1^) is the Langmuir constant. *R*_L_ indicates the shape of the isotherm, with 0 < *R*_L_ < 1 denoting favorable, *R*_L_ = 1 linear, *R*_L_ = 0 irreversible, and *R*_L_ > 1 unfavorable. In this study, MB adsorption onto FCNCs at all temperatures showed *R*_L_ values between 0 and 1, confirming favorable adsorption. The Langmuir model fit well (*R*^2^ = 0.9955), suggesting primarily monolayer adsorption on uniform sites, though slight deviations at high *C*_e_ (>800 mg L^−1^) hint at multilayer formation or dye aggregation. This is supported by the Freundlich heterogeneity parameter (1/*n* = 0.635), indicating moderate heterogeneity. Deviations may be due to π–π stacking, high surface area enabling multilayers, or hydrogen bonding after primary site saturation. Nonetheless, the process remains mainly monolayer-driven, as indicated by the high *R*^2^ and separation factor (0 < *R*_L_ < 1).

### 3.6 Comparison of MB dye adsorption of FCNCs with other CNCs

Table 3 compares the adsorption capacity of FCNCs derived from jute fiber utilizing APS oxidation with other CNCs documented in the current literature. The exceptionally high MB adsorption capacity of APS-oxidized cellulose nanocrystals (FCNCs), measuring 1566.95 mg g^−1^, is attributed to several factors. The one-pot APS oxidation procedure not only facilitates the separation of cellulose nanocrystals but also introduces a dense array of carboxylate groups at the C-6 position. Conductometric titration revealed 1470 mmol kg^−1^ of –COOH groups, indicating a theoretical monolayer binding capacity of approximately 1470 mg MB g^−1^, predicated on electrostatic attraction between –COO^−^ and MB^+^. The zeta potential measurement of −40 mV at pH 6.4 indicates a robust negative surface charge, which enhances the efficiency of electrostatic binding for positively charged dye molecules.

**Table 3:** Comparison among various CNCs and their adsorption capacity

Different adsorbent	Dye	Adsorption capacity (mg g^−1^)	Reference
CNCs	MB	94.43	37
CNCs	MB	101.16	38
Cellulose nanofibrils (CNFs)	MB	122.2	39
CNCs	MB	769	4
CNCs	MB	823	40
Electro-sterically stabilized nanocrystalline cellulose (ENCC)	MB	1400	6
FCNCs	MB	1566.95	This study

## 4. Conclusions

This study illustrated the synthesis and characterization of FCNCs derived from renewable, sustainable, and ubiquitous jute fibers by the environmentally friendly and cost-effective oxidization method and their application as an adsorbent for methylene blue dye. The FTIR analysis of FCNCs revealed a carbonyl peak at 1730 cm^−1^, confirming the oxidation process. The TGA exhibited good thermal properties, with a peak observed between 305 °C and 318 °C. A crystallinity index of 70% was recorded on the XRD graph, demonstrating that FCNCs are highly crystalline. The influence of several variables, such as temperature, initial dye concentration, and pH on dye uptake was studied. The Langmuir and the Freundlich isotherm models provided a good fit for the adsorption equilibrium data; however, the Langmuir model provided a more accurate description of the adsorption process. The highest possible adsorption capacity at room temperature and unadjusted PH (pH = 6.4) was 1566.95 mg dye per g FCNCs. The free energy change (Δ*G* = 0.545 kJ mol^−1^), enthalpy change (Δ*H* = −4.263 kJ mol^−1^), and entropy change (Δ*S* = −0.0158 kJ mol^−1^ K^−1^), among other calculated thermodynamic parameters, indicate that MB adsorption on FCNCs is a spontaneous exothermic process. Considering these results, FCNCs have the potential to be effective adsorbents for removing cationic dyes.

## Author contributions

Md. Fahimuzzaman contributed to the conceptualization, methodology, investigation, formal analysis, visualization, and writing of the original draft. Tarikul Islam contributed to the methodology, investigation, formal analysis, validation, and writing – review and editing. Md Shahabul Hossen contributed to the investigation, validation, visualization, and writing – review and editing. Khairul Islam contributed to the formal analysis, investigation, and writing – review and editing. Shanzida Binte Hassan contributed to the formal analysis, validation, and visualization. M. Mahbubul Bashar contributed to the conceptualization, methodology, formal analysis, resources, supervision, and writing – review and editing. All authors discussed the results and approved the final manuscript.

## Conflicts of interest

The author declared no conflicts of interest.

## Data Availability

Data will be available upon request from the corresponding authors.
